# Epidemiological Characteristics and Spatiotemporal Trend Analysis of Human Brucellosis in China, 1950–2018

**DOI:** 10.3390/ijerph17072382

**Published:** 2020-03-31

**Authors:** Huixin Yang, Siwen Zhang, Taijun Wang, Chenhao Zhao, Xiangyi Zhang, Jing Hu, Chenyu Han, Fangfang Hu, Jingjing Luo, Biao Li, Wei Zhao, Kewei Li, Ying Wang, Qing Zhen

**Affiliations:** 1 Jilin University School of Public Health, Department of Epidemiology and Biostatistics, Key Laboratory of Zoonosis Research, Ministry of Education, Changchun 130000, China; yhx18@mails.jlu.edu.cn (H.Y.); zhangsw18@mails.jlu.edu.cn (S.Z.); wangtj18@mails.jlu.edu.cn (T.W.); zhaoch2715@mails.jlu.edu.cn (C.Z.); xiangyi19@mails.jlu.edu.cn (X.Z.); hujing19@mails.jlu.edu.cn (J.H.); hancy2718@mails.jlu.edu.cn (C.H.); huff17@mails.jlu.edu.cn (F.H.); luojj17@mails.jlu.edu.cn (J.L.); libiao2717@mails.jlu.edu.cn (B.L.); 2 Jilin Provincial Center for Disease Control and Prevention, Microbiological laboratory, Changchun 130000, China; zyne2717@mails.jlu.edu.cn (W.Z.); lixl2717@mails.jlu.edu.cn (K.L.); 3 Jilin Province First Institute of Endemic Disease Control, Brucellosis Research Laboratory, Changchun 130000, China; yingw17@mails.jlu.edu.cn

**Keywords:** brucellosis, humans, epidemiology, zoonoses, China, GDP, spatiotemporal trend

## Abstract

The rate of brucellosis, a zoonotic disease, has rapidly increased in humans brucellosis(HB) in recent years. In 1950–2018, a total of 684,380 HB cases (median 2274/year (interquartile range (IQR) 966–8325)) were reported to the National Infectious Disease Surveillance System in mainland China. The incidence of HB peaked in 2014 (4.32/100,000), and then showed a downward trend; we predict that it will maintain a steady downward trend in 2019–2020. Since 2015, the incidence of HB has shown opposite trends in the north and south of China; rates in the north have fallen and rates in the south have increased. In 2004–2018, the most significant increases in incidence of HB were in Yunnan (IQR 0.002–0.463/100,000), Hubei (IQR 0.000–0.338/100,000), and Guangdong (IQR 0.015–0.350/100,000). The areas where HB occurs have little overlap with areas with high per capita GDP in China. The “high–high” clusters of HB are located in northeastern China (Inner Mongolia, Heilongjiang, Jilin, Liaoning, Ningxia, Shanxi, and Gansu), and the “low–low” clusters of HB are located in southern China (Yunnan, Jiangxi, Shanghai, Guangxi, Guangdong, Zhejiang, Guizhou, and Hunan). In recent years, the incidence of HB in China has been controlled to some extent, but the incidence of HB has increased in southern China, and the disease has spread geographically in China from north to south. Further research is needed to address this change and to continue to explore the relationship between the incidence of HB and relevant factors.

## 1. Introduction

Brucellosis is the most common zoonotic disease worldwide, with more than 500,000 new cases annually distributed in more than 170 countries. It represents a great risk to animal husbandry, with the potential to cause huge economic losses [1,2]. Brucellosis has become an important public health problem in China, and the number of HB cases has increased dramatically in the past few years [3]. The Communicable Disease Control Law of the People’s Republic of China stipulates that infectious diseases that must be managed are classified into three categories: Class A, Class B, and Class C. Among these, Class A diseases have been basically eradicated, so Class B diseases have become the focus of prevention and control of infectious diseases. The national epidemic report of infectious diseases in 2018 showed that a total of 7,770,749 infectious diseases were reported in that year, of which 3,063,021 were Class B infectious diseases. However, among the 23,377 deaths reported, 99.1% of the deaths were caused by Class B infectious diseases [4].

In July 1955, China’s “Measures for the Management of Infectious Diseases” listed brucellosis (wave fever) as a Class B infectious disease for reporting and management [5]. The epidemic situation of HB China was more serious in the 1950s and 1970s. It gradually declined in the 1980s and was basically controlled in the 1990s. However, the number of cases has surged since 2004, and reached a peak of 57,222 in 2014 [6,7].

Brucellosis in humans is mainly caused by exposure to brucella-infected livestock, aborted materials, or eating food that has not been pasteurized after being infected with brucella, especially dairy products of sheep and goats [8]. Epidemiological studies indicate that the pathogen that causes most HB worldwide is *Brucella melitensis* similarly, 84.5% of brucella strains isolated from patients with brucellosis in China are also *B. melitensis* [7]. Livestock including goats, cattle, and pigs are the main sources of brucellosis infection [9]. The main source of infection in China is sick sheep, followed by sick cattle and sick pigs. Brucellosis has a distinct seasonality; it can occur throughout the year, but the epidemic season is mainly in late spring and the entire summer. This seasonal high incidence is closely related to the lambing season. The peak season of sheep and lamb breeding in the vast pastoral areas of China is mostly in spring, which directly leads to herders having more opportunities to contact infected livestock [10,11,12]. In China, the first cases of brucellosis were discovered in 1905, with brucellosis occurring earlier in the north than in the south. The reason is that the north is a traditional pastoral area, and the development of animal husbandry in the south came much later than in the north. Nearly 90% of HB cases in China have appeared in five northern animal husbandry provinces: Inner Mongolia, Heilongjiang, Shanxi, Hebei, and Jilin [13]. However, in recent years, due to the introduction of northern cattle and sheep and other species, the HB epidemic is increasing in the southern provinces. It is worth noting that the incidence of HB in southern China is increasing. In 2012–2015, this growing trend was obvious in Guangdong, Zhejiang, Yunnan, Jiangsu, and Hunan [14].

In previous studies, a clear relationship between economic resources and health status has been observed across multiple countries, where developing countries with fewer resources are more susceptible to infections [15]. At present, many studies have demonstrated the relationship between per capita gross domestic product (GDP) and the incidence of Class B infectious diseases in China. Syphilis has a positive correlation with per capita GDP, tuberculosis has a negative correlation with per capita GDP, and hepatitis B and C, dysentery, and H1N1 influenza have nothing to do with economic development [16]. Brucellosis has been endemic to limited areas since its discovery. Thus, there is little literature on brucellosis nationwide, and little literature on its relationship with GDP. With the continuous improvement of China’s economic level since the 21st century, the mobility of people has greatly increased, and people are beginning to promote livestock activities in south China, especially raising pigs. Therefore, HB has become distributed in all provinces in China in recent years. Therefore, it is particularly important to investigate whether the occurrence of HB is related to economic factors.

In the past 15 years, HB cases in China have risen sharply. Related articles on the spatiotemporal characteristics of HB have also appeared, but we found that most of these articles were based on a province as the research unit, and provide limited guidance for national HB prevention and control [17,18,19,20]. There have been few studies to date that have used the latest data with a long span [6,21]. Simultaneously, we observed that most studies were retrospective and focused on surveillance and prevention of brucellosis rather than prediction. We used data on the incidence of HB from 1950 to 2018 to predict its incidence over the next two years, and described the distribution of HB and changes in its prevalence in mainland China using the notifiable reporting data for 2004–2018. At the same time, we performed a spatial autocorrelation analysis of brucellosis and showed the relationship between the geographical distribution of HB and GDP. Improving our understanding of HB epidemiology and changes in spatiotemporal distribution, and identifying high-risk areas can help to develop national strategic plans for the prevention and control of HB.

## 2. Method

### 2.1. Data Source

In this study, we used two datasets. One included the number and incidence of brucellosis cases by region from 1950 to 2013, provided by the National Health Cup Service Platform Public Health Scientific Data Center; the other was the number and incidence of brucellosis cases by age group and region from 2014 to 2018, provided by National Notifiable Infectious Disease Reporting Information System at the Chinese Center for Disease Control and Prevention.

The gross domestic product (GDP) data for the same period were obtained from the National Bureau of Statistics of China [22].

### 2.2. Ethical Considerations

China’s National Health and Family Planning Commission has determined that reporting data on HB cases is part of public health surveillance of legal infectious diseases, and does not need to be evaluated by the institutional review board. All data were provided and analyzed in an anonymous format with no access to personally identifiable information.

### 2.3. Statistical Analysis

Our analysis included all reported HB cases from 1 January 1950 through 31 December 2018. We use Holt–Winters exponential smoothing method to predict the number of cases in the following two years (2019–2020) with a CI of 95% on the basis of data reported in 2005–2018 [23]. We created a heat map of annual incidence to visualize the long-term changes in provinces in southern China over 15 years.

To provide information about the existence of the spatial correlations in the dependent variable, the global Moran’s I and Anselin’s local Moran’s I were correspondingly used for each year in the study area. The global Moran’s I index value was used to describe the global spatial autocorrelation among all 34 provinces. Local Moran’s I index was used to evaluate the correlation between individual target regions and the rest of the neighboring regions [24,25]. Local Moran’s I significance maps and cluster maps were used to display the four different types of local correlation and the significance of the corresponding Moran’s I index in various colors. High–high regions are geographical areas with a high incidence of brucellosis, surrounded by other geographical areas with high incidences of brucellosis. Similarly, low–low regions are geographical locations with low incidences of brucellosis, surrounded by other geographical areas with low incidences. High–high and low–low regions indicate clusters of geographic regions with similar brucellosis incidence values, while high–low and low–high regions indicate spatial outliers [26]. A Queen first-order continuity matrix was used to provide weights [27]. Moran’s I index was used to measure whether the disease distribution patterns were clustered or random [23,26]. In order to evaluate the importance of Moran’s I for the null hypothesis of spatial autocorrelation, we used a permutation process (randomization) of 999 on Open GeoDa free software to select the appropriate valid value. *p* < 0.05 was considered significant throughout.

Spearman’s correlation analysis was used to investigate the correlation between GDP and HB incidence between 2004 and 2018. Before conducting the Spearman’s correlation analysis, the values of the GDP and HB incidence were log-transformed. We used the average annual HB incidence times per capita GDP as a filter value to exclude provinces with a filter value of less than 100.

The above analysis was performed using Open GeoDa Environment 1.8.6 (Luc Anselin, Phoenix, AZ, USA) software. R statistical software (version 3.1.2, R Foundation for Statistics Computing, Vienna, Austria) was used with software package prediction (version 6.2) to generate heat maps and perform statistical analysis and prediction. Spearman’s correlation analysis was done using SPSS (version 24.0) software (IBM Corporation, Armonk, NY, USA), while ArcGIS 10.2.2 (ESRI, Redlands, CA, USA) was used for geographic patterns.

## 3. Results

### 3.1. Demographic Features

**Figure 1:**
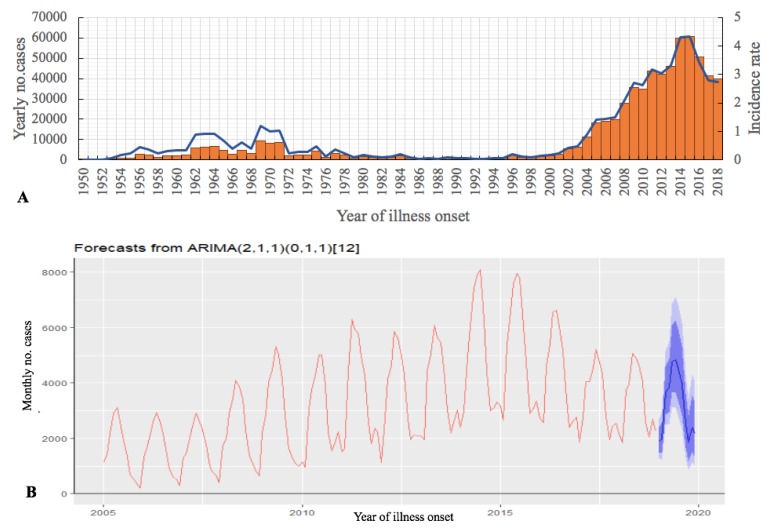
Human brucellosis (HB) cases (N = 684,380), mainland China, 1950–2018. (**A**) The number of cases reported by year (orange pillar) and annual incidence (blue line). (**B**) Autoregressive integrated moving average model (ARIMA) used to predict 2019–2020 data based on monthly numbers from 2005 to 2018, using 80% CI (light blue) and 95% CI (dark blue) monthly cases (blue line).

During the 1950–2018 period, the mainland China National HB Surveillance System reported a total of 684,380 HB cases (median 2274/year (interquartile range (IQR) 966–8325)) (Figure 1A). From 1950 to 2004, HB in China was in a low epidemic state. The incidence rate suddenly increased in 2004 (0.88/100,000), and reached a peak in 2014 (4.32/100,000). In 2018, the incidence rate dropped to 2.73/100,000.

We predicted that the incidence of HB nationwide will maintain a steady downward trend in the next two years (2019–2020): 38,360 (27,634–51,386) people will be affected in 2019 and 36,237 (18,639–57,909) people will be affected in 2020 (Figure 1B). In the report of infectious diseases published by the Chinese Center for Disease Control and Prevention, there were 46,700 cases of brucellosis from January to June 2019. We compared the number of reported cases in 2019 with the predicted value; the calculated model fit was 80.3%, and within the 95% confidence interval of the predicted value. The monthly forecast for 2019 to 2020 is shown in the Supplementary Materials.

### 3.2. Geographic Distribution

Previous studies have shown that brucellosis was present in northern China between 1950 and 1970, and that the incidence of the disease declined in the following 20 years [6]. However, in the mid-1990s, the disease reappeared, and the provinces in which the disease is endemic shifted from traditional livestock areas (such as Inner Mongolia, Qinghai Province, and Tibet) to inland areas (such as Shandong Province and Hebei Province).

**Figure 2:**
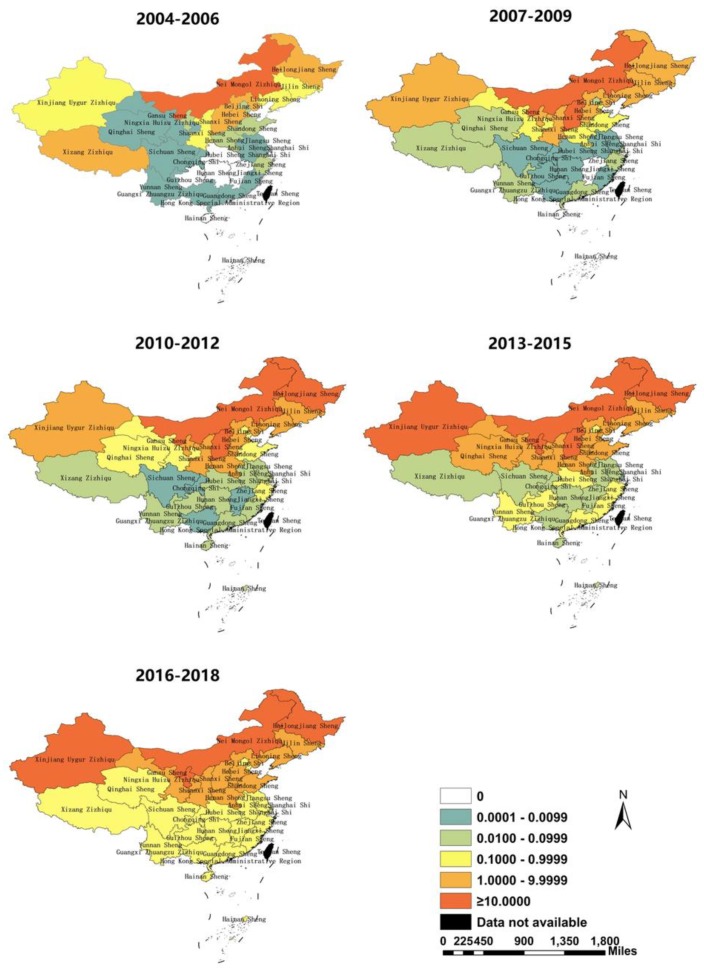
Annual incidence distribution of brucellosis in mainland China in 2004–2018 (by 3 year period).

In general, from 2004 to 2018, the prevalence of HB in China continued to expand, and the distribution of the disease gradually spread to the south. Every province in China has reported HB since 2010; the incidence of all provinces in southern China exceeded 0.100/100,000 in 2016–2018 (Figure 2). The three provinces with the highest incidence in 2004-2006 were Inner Mongolia (14.678/100,000), Heilongjiang (3.998/100,000), and Shanxi (3.678/100,000); in 2016–2018, the highest incidences shifted to Ningxia (26.716/100,000), Xinjiang (25.988/100,000), and Inner Mongolia (30.177/100,000). Meanwhile, since 2010, the incidence has increased in southern China, and HB has occurred or reappeared in all provinces in southern China.

**Figure 3:**
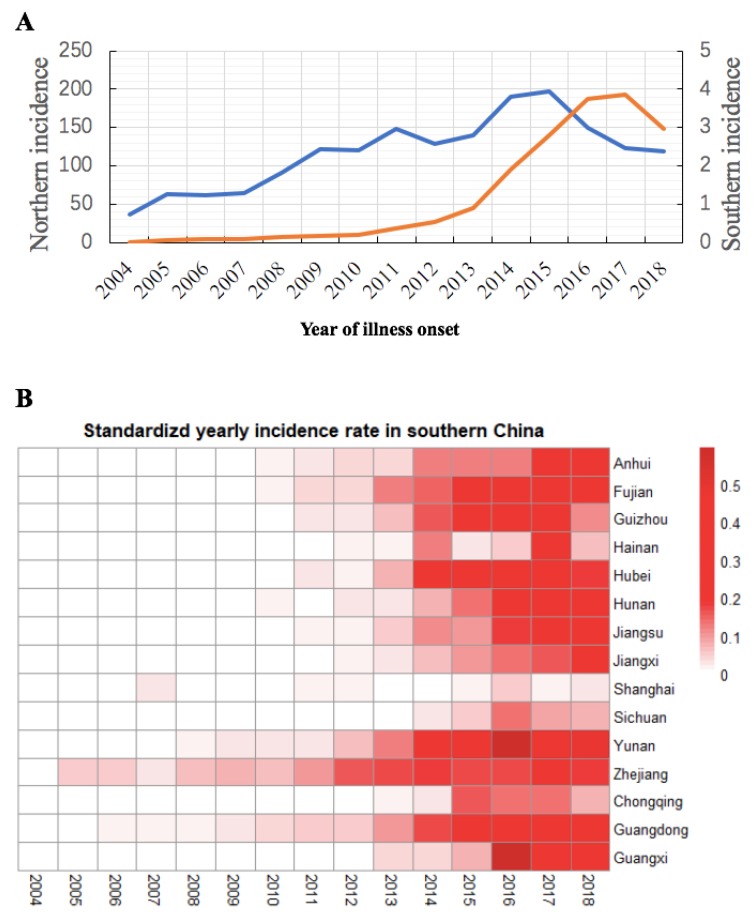
(**A**) The incidence (1/100,000) of HB in northern (blue line) and southern (orange line) China from 2004 to 2018. (**B**) Heat map of provinces with HB cases in southern China.

Leading up to 2015, the incidence of HB in northern China was on the rise, and it reached a 15 year peak in 2015 (197.73/100,000). After that, the incidence rate in northern China declined, from 2017 (122.85/100,000) flatten by 2018 (118.94/100,000). The incidence of HB in the south of China increased sharply in 2014 and reached a peak in 2017 (3.86/100,000) (Figure 3A).

The annual incidence rate of HB fluctuated in southern China over the 15 years studied (Figure 3B). Before 2010, except for Guangdong, Zhejiang, Yunnan, Hunan, and Fujian Provinces, there was almost no HB in southern China. According to reports, HB occurred in all provinces in southern China in 2012. In 2004–2018, the most significant increases were in Yunnan (IQR 0.002–0.463/100,000), Hubei (IQR 0.000–0.338/100,000), and Guangdong (IQR 0.015–0.350/100,000). In 2018, the top three provinces with the highest incidences of HB in southern China were Yunnan (0.479/100,000), Guangdong (0.352/100,000), and Guangxi (0.276/100,000).

### 3.3. Brucellosis and GDP per Capita

**Figure 4:**
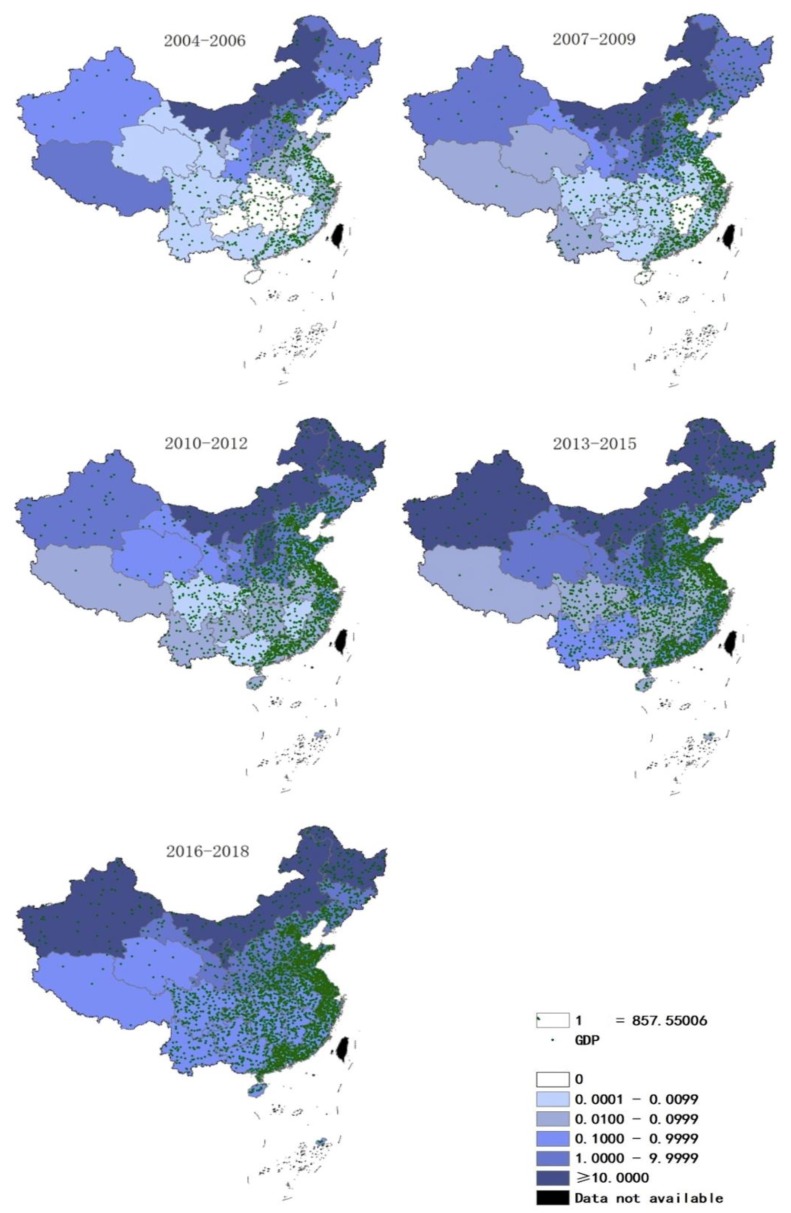
Annual incidence and gross domestic product (GDP) (/100 million yuan) distribution in mainland China, 2004–2018 (by 3 year period).

The GDP in southeast coastal regions is higher than that in inland regions. From 2004 to 2018, Guangdong Province (80,329.2 billion yuan), Jiangsu Province (74,220.8 billion yuan), and Shandong Province (67,522.8 billion yuan) were the top three provinces by GDP (Supplementary Material). Between 2004 and 2018, fewer cases of HB occurred in the southern provinces with higher GDP than in the northern regions (Figure 4).

Through Spearman’s correlation analysis, we found that the correlation between per capita GDP levels and the incidence of HB in 2016–2018 was negative (Table 1). In other time periods, although the *p*-value was not statistically significant, the correlation coefficients were all negative. This was similar to the result of another recently published paper [21].

### 3.4. Distribution of Four Different Clusters

**Figure 5:**
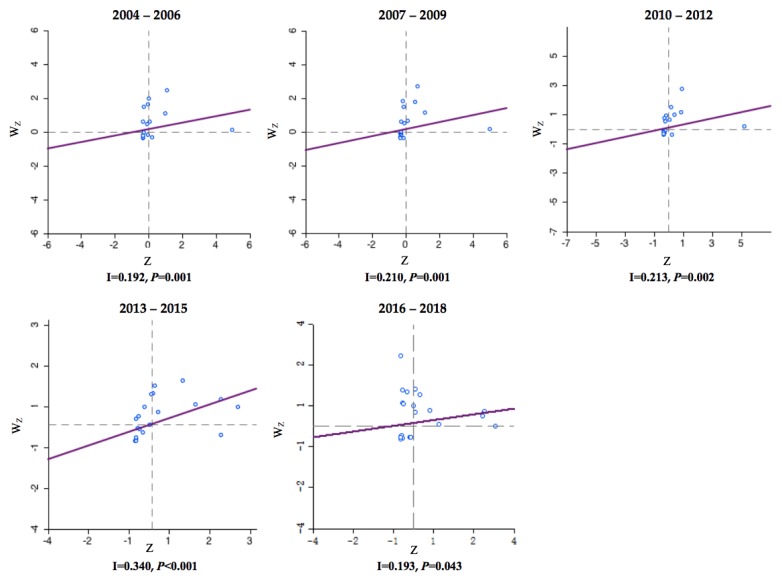
The Moran’s I scatter plots of the annual incidence of HB in China from 2004 to 2018 (by 3 year period). The first to fourth quadrants of the Moran scatter plot correspond to high–high, low–high, low–low, and high–low correlations of local Moran’s I. The horizontal axis of the Moran scatter plot is the observed and normalized z-score ($Z_{i}=\frac{X_{i}-X}{S}$) for a specific province or district, and the vertical axis is the weighted sum of observed and normalized z-score for the neighboring provinces or districts ($W_{Z_{i}}=\overset{n}{\underset{i=1}{\sum }}w_{ij}z_{j}$ ). The individual dots represent the specific 34 provinces or districts.

**Figure 6:**
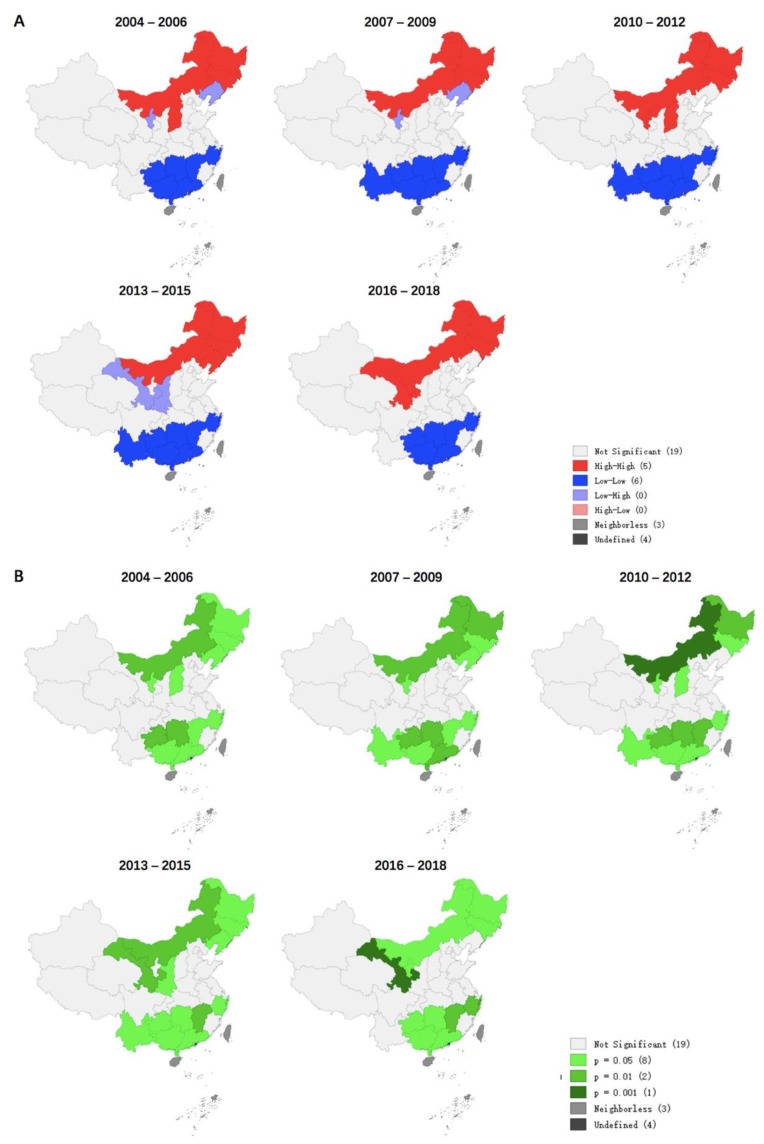
Moran’s I chart of HB incidence rate per 100,000 residents in China from 2004 to 2018 (by 3 year period). (**A**) Local Moran’s I cluster map. (**B**) Local Moran’s I significance map.

Between 2004 and 2018, the incidence of HB in each county or region showed significant spatial autocorrelation and spatial clustering, with a global Moran’s I index ranging from 0.192 to 0.340 (*p* < 0.05). Most cluster points in the Moran scatter plot were in the upper right and upper left quadrants, with the most points observed in the upper left quadrants and the fewest points observed in the lower right quadrants (Figure 5). Four different types of local spatial autocorrelation clusters appeared simultaneously and were visualized by local Moran’s I cluster map (Figure 6A) and saliency map (Figure 6B).

Combining spatial autocorrelation and Moran scatter plot, we found that the main clusters were low–low clusters, followed by high–high clusters, and high–low clusters were the least frequent. Low–low clusters were mainly concentrated in the provinces of southern China (Yunnan, Jiangxi, Shanghai, Guangxi, Guangdong, Zhejiang, Guizhou, and Hunan); high–high clusters were concentrated in four provinces in northern China (Inner Mongolia, Heilongjiang, Jilin, Liaoning, Ningxia, Gansu, and Shanxi); Ningxia, Liaoning Gansu, and Shaanxi were low–high clusters. In China, there were no high–low clusters of HB.

## 4. Discussion

We used China’s longitudinal monitoring dataset from 1950 to 2018 to describe the epidemic characteristics and the changes of spatiotemporal trend of HB, especially during the period of socioeconomic change in the past 15 years. We predicted a decline in the incidence of HB in 2019 and 2020, contrary to the conclusions of Lai et al. [6]. The actual number of cases in 2019 was found to show a slight upward trend, but it was still within our forecast range, and there was no exponential upward trend as indicated by other literature. We think that this was caused by the different time range of the basic dataset used. Considering the effect of time interval on the development of data, the exponential smoothing method used in this paper is usually used to predict short-term disease incidence; therefore, we used the incidence data of 2005–2018 to predict the incidence of 2019–2020, which guaranteed the high accuracy of the predicted values [23].

The prevalence of brucellosis in humans mainly depends on the epidemic situation in animals, and the changes in its distribution range identified in our article are consistent with the epidemic law in China [6,10,28]. From the mid-1970s to 1990, China formulated and implemented a number of policies to control brucellosis and achieved good results [7,29,30]. In the same period, the pasteurization technology of China’s dairy industry developed greatly; by the late 1990s, pasteurized or ultra-high-temperature sterilized milk was being sold from the north to all parts of the country [31]. The continuous maturation of sterilization technology has played a role in reducing the incidence of HB during this time. However, the epidemic has been fighting back since the late 1990s, mainly for the following reasons.The first reason is the persistence of the source of infection; the diseased animals in the old epidemic pastoral areas have not been completely removed. At the same time, sick animals found through quarantine inspection cannot be properly handled due to unacceptable or unsatisfactory compensation funds for farmers [32,33]. Secondly, the country’s increasing demand for cattle, sheep, and other livestock has also led to an increasing incidence of HB [11,12]. The plan made by the National Development and Reform Commission is that by 2020, the proportion of large-scale beef cattle breeding in the three northeastern provinces will account for 55% of the country’s stock, and sheep breeding in the western region will reach 45% [34]. Thirdly, the combined use of multiple diagnostic methods for brucellosis is also one of the reasons for the rising incidence of reported brucellosis in humans. With the rapid development of molecular biology technology since the 1990s, molecular biological detection methods have been used to rapidly detect brucellosis at home and abroad [35,36,37]. For example, studies have shown that multiple real-time polymerase chain reactions can diagnose patients with negative serological tests, which makes up for the shortcomings of traditional serological tests and is considered an important detection method [38].

Our research showed that the reported incidence of HB nationwide in 2016 decreased by 21.6% compared with 2015, which was the first time that the incidence of brucellosis cases had fallen since 2010. It continued to decline in 2017 to an even greater degree, down 23.7% from 2016. This trend had different characteristics in the north and south. The incidence in the north has decreased, while the incidence in the south has continued to rise. The main reason for the decline in HB in northern China in recent years is that the key northern epidemic areas have paid more and more attention to brucellosis, such as the establishment of a provincial epidemic monitoring network, the implementation of a vaccine immunization approval system, and a diseased animal culling system [39,40,41]. In our research, we found that HB has spread and increased in southern China over nearly 15 years. The important finding is that the areas in southern China with low incidences of HB, such as Hubei, Yunnan, Guangxi, and Guangdong, show an increasing trend, which is consistent with the conclusions of multiple studies [14,42,43]. In addition, Lai et al. [6]. also described the change in the incidence of brucellosis in southern China in their article, but the study time span was different from that analyzed in this article. In order to supplement and extend the results, we followed the trend of HB in north and south China from 2004 to the present. Compared with their conclusion that HB was circulating in south China from 1955 to 2014, our results indicated that the incidence of HB in South China has increased significantly, and it is rising rapidly. At the same time, we noticed that over 10 years of HB transmission, the distribution of pathogenic species of brucellosis in China changed dramatically. Since the 1950s, *B. melitensis* has been most common on grasslands in northern China, where sheep and goats are the main livestock [6,10,20]. The pathogens of brucellosis appearing in southern China also include *B. abortus* or *B. suis* [44,45]. Since 2000, there have been continuous studies showing that the seroprevalence of brucellosis in sheep/goats and cattle in the southern part of the country is rising, especially in the provinces of Chongqing, Guizhou, and Sichuan [46].

There are many reasons for the increase in the number of HB cases in southern China. First, in some cases, sick sheep and their products that have not been quarantined in the north have entered the south, further continuing the HB epidemic. With the rapid development of transportation, economy, and the Internet, frequent livestock and product transactions are now occurring between the north and the south. In addition, some studies have shown that increased numbers of livestock raised by local retail farmers in the south, insufficient quarantine immunity, irregular slaughter, and inadequate treatment of infectious sources may also be increasing the epidemic intensity of the disease [47,48]. In addition to expanding animal husbandry in southern China, changes in dietary structure have increased the risk of more people coming into contact with raw lamb or beef (such as hot pots, boiling water, or soups). What requires attention is the potential impact of awareness on infection rates. All the results of multiple knowledge surveys conducted in the major endemic areas of brucellosis in China show that people’s awareness of the disease is now much higher than it was before [49,50,51]. This will cause a considerable number of people to pay attention to protection and infection, and early symptoms can be noted in time for medical treatment. However, in most provinces in southern China, the related occupational populations have relatively weak awareness of brucellosis. It is important to reduce the incidence of HB in southern China, strengthen quarantine and management of agricultural markets, and manage domestic transportation of livestock to prevent contaminated meat and its products from entering the market. At the same time, receiving occupational disease inspections, avoiding direct contact with animal product in epidemic areas, and providing workers with appropriate health education are also important in avoiding occupational infections [52,53].

Our research showed that in China, the distribution of regions with high incidence of HB had almost no overlap with the distribution of high levels of per capita GDP. This was consistent with the conclusions of many previous studies [15,21]. A global study showed that countries with relatively high per capita GDP tend to have less brucellosis; another study showed that rainfall and per capita GDP in China’s temperate regions are low and negatively related to the number of cattle and sheep. Therefore, the prevalence of HB in China’s moderate temperate zone (most of the northeast region, the Inner Mongolia Plateau) is very high. At the same time, some people believe that socioeconomic status is related to the increasing trend of brucellosis infection, and that northern China is more affected by HB [42]. The reason may be that sanitary conditions are generally poor in areas with poor economic conditions, which promotes the spread of foodborne and soil-borne diseases [54]. Moreover, China’s economy has entered a period of rapid growth, with per capita GDP increasing from US$1828 in 2004 to US$8828 in 2017 [55]. With the rapid acceleration of urbanization in developing countries, the emergence of infectious diseases in traditional pastoral areas and rural areas also poses a major threat to urban residents [54]. In addition, the increasing living standards of the population have stimulated the demand for meat, which has led to an increase in the demand for animal husbandry and animal products [56]. However, there are still studies showing that in the subtropical region of China (southern China), the number of cattle and sheep, precipitation, and GDP per capita have hardly affected the occurrence of HB [21]. According to the results of our current research, we cannot give a direct reason for this observation. The relationship between the incidence of HB, the per capita GDP level, and other environmental factors still needs to be explored further in order to formulate comprehensive prevention and control measures for HB.

According to our analytical data, the spatial autocorrelation analysis showed significant spatial clustering of HB incidences in China between 2004 and 2018 but varied by different provinces. the “high–high” clusters of HB were located in northeastern China, while the “low–low” clusters were located in southern China. First, the reasons for the development of local animal husbandry, which plays a vital role in the incidence of HB, should be considered. The areas where HB is highly concentrated have similar natural and social environments, which may also increase the risk of HB epidemics in these areas [6,21,39,57]. Another potential reason for the spatial distribution of HB is the relative economic disadvantage of the high–high districts. Relevant research shows that herdsmen’s educational level is generally low, and that they lack basic knowledge about the transmission of HB and its prevention and control measures [1,58,59]. Bad habits such as unprotected delivery of lambs, cleaning of sheep pens, drinking raw goat milk, and eating raw meat can also greatly increase the risk of HB [60]. In addition, herdsmen’s related protective equipment (masks, gloves) in underdeveloped areas are inadequately purchased, and they are more likely to be exposed to brucellosis [61]. At the same time, since the initial symptoms of brucellosis infection are similar to colds (such as fever, weakness in the limbs), many farmers and herdsmen do not seek medical treatment soon enough after infection [9]. In contrast, low–low clusters, including Shanghai and Guangdong, generally have economic advantages, and their incidence of HB is relatively low. The low–high concentration areas should also be paid attention; these areas indicate a lower incidence in some areas and higher values in other provinces bordering it. Adjacent areas with high incidence should prompt strengthening of quarantine and management of related animals in order to prevent the spread of diseases. In short, in view of the geographical distribution of HB, related public health issues should be given priority in northern China. Although most of the south is in a low-concentration area, it is related to the size of the geographic unit studied. Potential high-value clusters may exist in the southern region, which requires further narrowing of geographic units for research.

To sum up, based on China’s large-scale, population-based surveillance system, we compiled HB report data for 69 consecutive years (1950–2018), providing us with strong support for the reliability of the conclusions and the prediction of accuracy. A comprehensive study of the overall spatial distribution pattern of HB in China was conducted using geographic information systems, and its relationship with the per capita GDP level was explored.

## 5. Conclusions

Our research provides scientific evidence for the development and modification of prevention and control strategies for the HB epidemic in China. First of all, it is necessary to increase the investment of funds and health resources in northern China, strengthen the education of HB prevention and control knowledge, and in addition, to strictly control the inter-provincial spread of HB and avoid large-scale infection of HB in southern China. The transmission of information between northern and southern medical staff should be strengthened to prevent misdiagnosis and missed diagnosis due to insufficient knowledge of HB in the south. Moreover, the distribution of the incidence of brucellosis is different from the level distribution of per capita GDP. Further research is needed to explore the drivers of epidemiological change of HB and their relationship with per capita GDP.

## Figures and Tables

**Table 1:** Spearman’s correlation coefficients between HB incidence and GDP from 2004 to 2018 (by 3 year period).

Time Period	r	*p*
2004–2006	−0.336	0.312
2007–2009	−0.274	0.272
2010–2012	−0.286	0.148
2013–2015	−0.224	0.233
2016–2018	−0.360	0.047

## Supplementary Materials

The following are available online at https://www.mdpi.com/1660-4601/17/7/2382/s1, Table S1: Time series predictions of human brucellosis in the months of 2019–2020., Table S2: Per capita GDP distribution of each province in mainland China, 2004–2018 (by three years) (/100 million yuan).
