# Supplementary material for: Epidemiological Characteristics and Spatiotemporal Trend Analysis of Human Brucellosis in China, 1950–2018
Source: Int J Environ Res Public Health. 2020 Mar 31;17(7):2382. doi: 10.3390/ijerph17072382 (PMC7178157; doi:10.3390/ijerph17072382)
Supplement: Supplementary file 1 [file ijerph-17-02382-s001.pdf]

Table S1 . Time series predictions of human brucellosis in the months of 2019-2020

Year	Month	Point forecast	Low 80	High 80	Low 95	High 95
2019	Jan	1902.133	1469.304	2412.001	1269.247	2715.203
2019	Feb	1965.608	1463.486	2570.381	1236.031	2935.274
2019	Mar	3648.835	2846.250	4588.659	2473.312	5145.349
2019	Apr	3824.242	2933.482	4877.997	2523.331	5506.383
2019	May	4770.822	3656.139	6090.227	3143.146	6877.322
2019	Jun	4841.119	3654.494	6258.529	3112.879	7109.140
2019	Jul	4506.417	3324.510	5937.809	2791.827	6804.522
2019	Aug	3941.756	2817.720	5328.820	2319.891	6178.806
2019	Sep	2518.938	1666.251	3619.904	1304.727	4313.827
2019	Oct	1872.948	1158.255	2831.835	866.727	3450.281
2019	Nov	2391.551	1513.570	3554.212	1150.619	4298.285
2019	Dec	2175.229	1130.332	3315.912	987.905	4054.320
2020	Jan	1798.755	966.188	3005.826	653.697	3819.641
2020	Feb	1817.947	931.894	3134.980	608.617	4035.158
2020	Mar	3445.720	1984.338	584.751	1412.566	6832.158
2020	Apr	3629.616	2048.453	5861.426	1437.172	7343.710
2020	May	4536.441	2603.456	7239.940	1848.710	9026.168
2020	Jun	4600.847	2580.841	7461.563	1802.722	9365.114
2020	Jul	4280.064	2303.775	7142.140	1561.077	9070.578
2020	Aug	3735.306	1894.760	6486.297	1227.525	8372.225
2020	Sep	2365.392	1020.626	4549.233	580.023	6112.846
2020	Oct	1747.015	653.932	3649.391	327.461	5058.846
2020	Nov	2243.356	893.190	4524.487	473.207	6190.348
2020	Dec	2036.143	757.289	4268.615	376.956	5925.078

Low80-High80 represents 80% confidence interval; Low95-High95 represents 95% confidence interval

Table S2. Per capita GDP distribution of each province in mainland China, 2004-2018 (by three years) (/ 100 million yuan)

districts	2004-2006	2007-2009	2010-2012	2013-2015	2016-2018	Over all
Beijing	21120.51	33114.84	48244.91	64146.23	84004.05	250630.54
Tianjin	11479.35	19493.62	33425.62	46707.13	55244.22	166349.94
Hebei	29957.34	46854.77	71485.03	87670.21	102097.04	338064.39
Shanxi	12680.51	20698.16	32551.24	38193.23	45396.94	149520.08
Inner Mongolia	11890.35	24659.63	41912.46	52518.2	51513.53	182494.17
Liaoning	24023.78	40045.37	65530.4	84508.82	70971.49	285079.86
Jilin	11017.4	18989.54	31175.65	40912.67	44795.95	146891.21
Heilongjiang	16476.1	24005.37	36642.18	44577.96	47650.39	169352
Shanghai	27892.73	41610.32	56543.39	70509.3	91491.51	288047.25
Jiangsu	55344.34	91457.76	144593.97	194958.07	255853.44	742207.58
Zhejiang	40784.85	63206.77	94706.49	120816.11	155216.77	474730.99
Anhui	16221.97	26275.4	44872.03	62083.72	81432.44	230885.56
Fujian	19901.89	32308.07	51999.08	71904.07	96796.71	272909.82
Jiangxi	12333.99	20426.48	34102.96	46848.6	60490.09	174202.12
Shandong	55288.9	90606.84	134545.01	177659.24	217128.31	675228.3
Henan	31504	52511.45	79622.7	104131.7	133080.48	400850.33
Hubei	19840.9	33623.42	57850.32	81721.24	107510.02	300545.9
Hunan	19926.71	34054.29	57861.75	80561.2	101880.11	294284.06
Guangdong	68009.75	108056.28	156291.26	203097.19	267837.91	803292.39
Guangxi	12163.76	20603.57	34325.82	46925.91	57193.41	171212.47

districts	2004-2006	2007-2009	2010-2012	2013-2015	2016-2018	Over all
Hainan	2804.08	4411.44	7442.7	10381.04	13347.79	38387.05
Chongqing	10409.53	16999.8	29346.55	42763.13	57528.51	157047.52
Sichuan	22454.97	37314.9	62084.96	84981.83	110592.89	317429.55
Guizhou	6022.2	10358.35	17156.2	27855.81	40124.01	101516.57
Yunnan	10532.78	16634.39	26426.77	38266.07	49045.88	140905.89
Tibet	759.9	1177.64	1814.32	2762.89	3939.96	10454.71
Shannxi	11852.91	21241.67	37089.46	51917.25	65736.72	187838.01
Gansu	5899.82	9258.36	14791.32	19957.83	22906.34	72813.67
Qinghai	1657.92	2897.24	4914.41	6842.43	8062.55	24374.55
Ningxia	1875.62	3476.34	6133.15	8241.44	10317.33	30043.88
Xinjiang	7858.54	11983.42	19552.83	27042.1	32730.74	99167.63

Continue for table S2.
